# Erratum for Barbosa Bomfim et al., “CGRP inhibits SARS-CoV-2 infection of bronchial epithelial cells, and its pulmonary levels correlate with viral clearance in critical COVID-19 patients”

**DOI:** 10.1128/jvi.02006-24

**Published:** 2024-12-10

**Authors:** Caio César Barbosa Bomfim, Hugo Génin, Andréa Cottoignies-Callamarte, Sarah Gallois-Montbrun, Emilie Murigneux, Anette Sams, Arielle R. Rosenberg, Sandrine Belouzard, Jean Dubuisson, Olivier Kosmider, Frédéric Pène, Benjamin Terrier, Morgane Bomsel, Yonatan Ganor

## ERRATUM

Volume 98, no. 9, e00128-24, 2024, https://doi.org/10.1128/jvi.00128-24. Olivier Kosmider’s name should appear as shown in this erratum.

